# A Small Library of Synthetic Di-Substituted 1, 4-Naphthoquinones Induces ROS-Mediated Cell Death in Murine Fibroblasts

**DOI:** 10.1371/journal.pone.0106828

**Published:** 2014-09-08

**Authors:** Oscar Ramirez, Laura B. Motta-Mena, Amanda Cordova, Kristine M. Garza

**Affiliations:** 1 Dept. of Biological Sciences, University of Texas at El Paso, El Paso, Texas, United States of America; 2 Border Biomedical Research Center, University of Texas at El Paso, El Paso, Texas, United States of America; University of Colorado, Denver, United States of America

## Abstract

Synthesis of compound libraries and their concurrent assessment as selective reagents for probing and modulating biological function continues to be an active area of chemical biology. Microwave-assisted solid-phase Dötz benzannulation reactions have been used to inexpensively synthesize 2, 3-disubstituted-1, 4-naphthoquinone derivatives. Herein, we report the biological testing of a small library of such compounds using a murine fibroblast cell line (L929). Assessment of cellular viability identified three categories of cytotoxic compounds: no toxicity, low/intermediate toxicity and high toxicity. Increased levels of Annexin-V-positive staining and of caspase 3 activity confirmed that low, intermediate, and highly toxic compounds promote cell death. The compounds varied in their ability to induce mitochondrial depolarization and formation of reactive oxygen species (ROS). Both cytotoxic and non-cytotoxic compounds triggered mitochondrial depolarization, while one highly cytotoxic compound did not. In addition, all cytotoxic compounds promoted increased intracellular ROS but the cells were only partially protected from compound-induced apoptosis when in the presence of superoxide dismutase, catalase, or ascorbic acid suggesting utilization of additional pro-death mechanisms. In summary, nine of twelve (75%) 1, 4-naphthoquinone synthetic compounds were cytotoxic. Although the mitochondria did not appear to be a central target for induction of cell death, all of the cytotoxic compounds induced ROS formation. Thus, the data demonstrate that the synthesis regime effectively created cytotoxic compounds highlighting the potential use of the regime and its products for the identification of biologically relevant reagents.

## Introduction

Quinones are aromatic compounds naturally present in bacteria and eukaryotes. They are often involved in the biochemistry of energy production and serve as vital links in electron transport in the form of ubiquinones [1]. This biological activity is related to the acceptance of one and/or two electrons to form the corresponding radical anion or dianion species (electrophiles). Quinones are also natural defensive products made by plants and have been employed as anti-fungal agents, broad-spectrum anti-bacterials, and anti-malarial drugs [2]–[4]. Moreover, extensively substituted anthroquinones or *p*-benzoquinones or naphthoquinones with reactive or heterocyclic groups are effective anti-cancer agents [5], [6] forming one of the largest classes of cytotoxic agents used therapeutically against cancer.

Quinones are particularly effective at inducing apoptosis [7], [8] and as such provide a rich source of unique cytotoxic reagents that can be exploited. Of note, are the anti-malarial naphthoquinones, particularly hydroxyl-1, 4-naphthoquinone (atavoquone), which is used in combination with proguanil (known as Malarone) for the prevention and treatment of malaria [9], [10]. In an effort to create 1, 4-naphthoquinone libraries a novel organic synthesis regime was developed by Shanmugasundaram et. al using microwave-assisted solid-phase Dötz benzannulation reactions [11]. This was the first report to use solid-supported Dötz benzannulation reaction and the subsequent oxidative cleavage process to generate derivatives of this class of quinones.

Here, twelve different 2, 3-disubstituted-1, 4-naphthoquinones synthesized using this regime were screened for biological activity against the murine fibroblast cell line, L929. The L929 cell line was chosen to serve as the adherent cell assay model due to its ease of use and its frequent use in toxicity assays for various agents [12]–[14]. The data demonstrates that the majority of the naphthoquinones studied were cytotoxic and promoted the induction of ROS formation.

## Materials and Methods

### Compounds

2, 3-disubstituted naphthoquinones were synthesized on solid support utilizing the Dotz reaction with solid supported Fischer carbine complexes as described [11]. The compounds were analyzed by gas chromatography and ranged from 95–99% purity. They were dissolved in DMSO at 20 mg/ml and stored frozen at −20°C.

### Cell culture

The murine fibroblast cell line, L929, was purchases from ATCC (NCTC clone 929; L cell, L-929 derivative of Strain L; ATCC CCL-1). The cells were grown in Dulbecco's Modified Eagles Medium (DMEM) supplemented with 10% fetal bovine serum, 100 Units/ml Penicillin, 100 µg/ml Streptomycin, and 1X Glutamax (all cell culture reagents were purchased from Invitrogen Corp., Grand Island, NY). The cells were grown at 37°C, 5% CO_2_ and were used for experiments when in expansion phase of culture (at approximately 70% confluency).

### MTT viability assay

The MTT (3-(4, 5-dimethylthiazol-2-yl)-2, 5-diphenyl tetrazolium bromide) viability assays were carried out as per manufacturers' instructions (Sigma). The L929 cells were plated at 50,000 cells per well in a 96-well flat-bottom plate (Becton-Dickinson Labware, Franklin Lakes, NJ). The cells were cultured with decreasing concentrations of the compounds (in duplicate), beginning at 0.50 mg/ml and decreasing by half over eleven additional dilutions. As controls, cells remained untreated (media alone) or were treated with equivalent dilutions of dimethylsulfoxide (DMSO, vehicle control) (Sigma). Following a 48 h incubation, the MTT reagent was added at 10% of the total volume per well. Following a 4 h incubation, the formed crystals were solubilized by removing approximately ¾ of the supernatant and adding 50 µl of solvent (10% Triton × and 1% 12 N HCl in isopropanol) (Sigma). All wells were vigorously pipetted (without forming bubbles) to help dissolve the crystals. The plates were then read in a 96-well plate spectrophotometer at 570 nm.

### Microscopy of compound-treated cells

Cells were grown at a density of 1×10^5^ cells per well in a four-well chambered slide (Nalgene Nunc, Rochester, NY) in the presence of medium alone, the indicated compound (IC_50_), DMSO, or H_2_O_2_. The cells were cultured for 24 h at 37°C, 5% CO_2_. After the 24 h incubation, the cells were observed at 40× using a Zeiss Axiovert 200 microscope. Digital images were captured using the AxioCam HR digital camera and were processed with Axiovision Software, Version 4.1.

### Flow cytometry assessment of apoptosis

L929 cells (500,000 cells/well) were cultured in 12-well plates for 48 h in the presence of medium, DMSO, or compound (IC_50_). The cells were then washed in FACS buffer (phosphate buffered saline +2% fetal bovine serum), resuspended in 50 µl of buffer and then incubated with 30% normal mouse serum (Sigma) to prevent non-specific staining, for 15 min. at room temperature. The cells were then treated with 5 µl of FITC-conjugated annexin V (1 mg/ml stock) and 2 µl of propidium iodide (5 mg/ml stock). The cells were then mixed gently and incubated at room temperature for 15 minutes in the dark followed by an analysis by flow cytometry within the hour. 10,000 events per sample were collected and analyzed by EXPO32 software.

### Detection of active caspase-3

The assay was performed as per the manufacturers' instructions (BioSource International, Camarillo, CA). The L929 cells were plated at 3×10^6^ cells per well in 6-well plates. The cells were then treated with the indicated compounds for 24 h. After treatment with the compounds, the cells were centrifuged, washed twice with 1× PBS, were re-suspended in 50 µL of chilled Cell Lysis Buffer and were incubated for 10 min. on ice. The samples were then centrifuged and the cytosolic extracts were then transferred to 96-well flat-bottom plates. Reaction buffer (5 µL of 1.0 M DTT to 500 µL of 2× Reaction buffer), made just prior to use, and was added to each well. DEVD-pNA substrate (5 µL) was then added to each well and the samples were incubated in the dark for an additional 2 h. The plates were then read on a spectrophotometer at a wavelength of 405 nm.

### Detection of mitochondrial membrane depolarization

L929 cells (5×10^4^ cells/well) were cultured in 12-well plates for 48 h in the presence of medium, DMSO, camptothecin (4 µM, positive control) or compound (IC_50_). The cells were then washed in FACS buffer resuspended in 100 µl of buffer. DePsipher (5, 5′6, 6′-tetrachloro-1, 1′, 3, 3′-tetraethylbenzimidazolylcarbocyanine iodide) (5 µl per sample) (Trevigen, Inc., Gaithersburg, MD) was then added. The cells were incubated at room temp., in the dark for a minimum of 15 min. The cells were assayed within the hour by flow cytometry. 10,000 events per sample were collected and analyzed by EXPO32 software.

### Detection of reactive oxygen species (ROS) by DHCF-DA

Cells were plated at 50,000 cells per well in 96-well plates, protected or unprotected by 10 µM ascorbic acid. Following a 4 h incubation period, the cells were then treated with the indicated compounds at the IC_50_ or with H_2_O_2_ as a positive control. Twenty-four hours later, the plates were centrifuged and the supernatants were collected. The adherent cells were treated with 20 µL of 0.25% trypsin (Invitrogen) for 2 min. The cells were then removed and transferred to black-walled 96-well plates to prevent bleeding of fluorescence between sample wells. Dichlorofluorescein-Diacetate (10 µM) (Invitrogen - Molecular Probes), was then added to each well. The plates were then immediately read on a fluorescent plate reader using excitation wavelength of 485 nm and emission wavelength of 538 nm. Fluorescence was determined every 10 min over a 320 min. period.

### Ascorbic acid protection assay

Prior to the viability assay, the cells were treated for 24 hrs with 10 µM ascorbic acid (Sigma). The cells were then thoroughly washed with Hank's Balanced Salt Solution (HBSS (Invitrogen) and the MTT viability assay was conducted as described above.

### Catalase and super oxide dismutase (SOD) protection assay

L929 cells were plated at 50,000 cells/well in a 96-well flat-bottom plate. Catalase (1 mg/ml), SOD (0.5 mg/ml), or combinations of the two were added to the cells. The cells were also treated with the indicated compounds at a concentration that inhibited 50% of cellular viability or with media or DMSO as controls. Following a 48 h incubation period, cellular viability was assessed as described above (MTT assay).

### Statistical analysis

Statistical significance was analyzed with Student's t-test. The level of significance was set at 0.05.

## Results

### Compound effects on cellular viability and morphology

**Figure 1:**
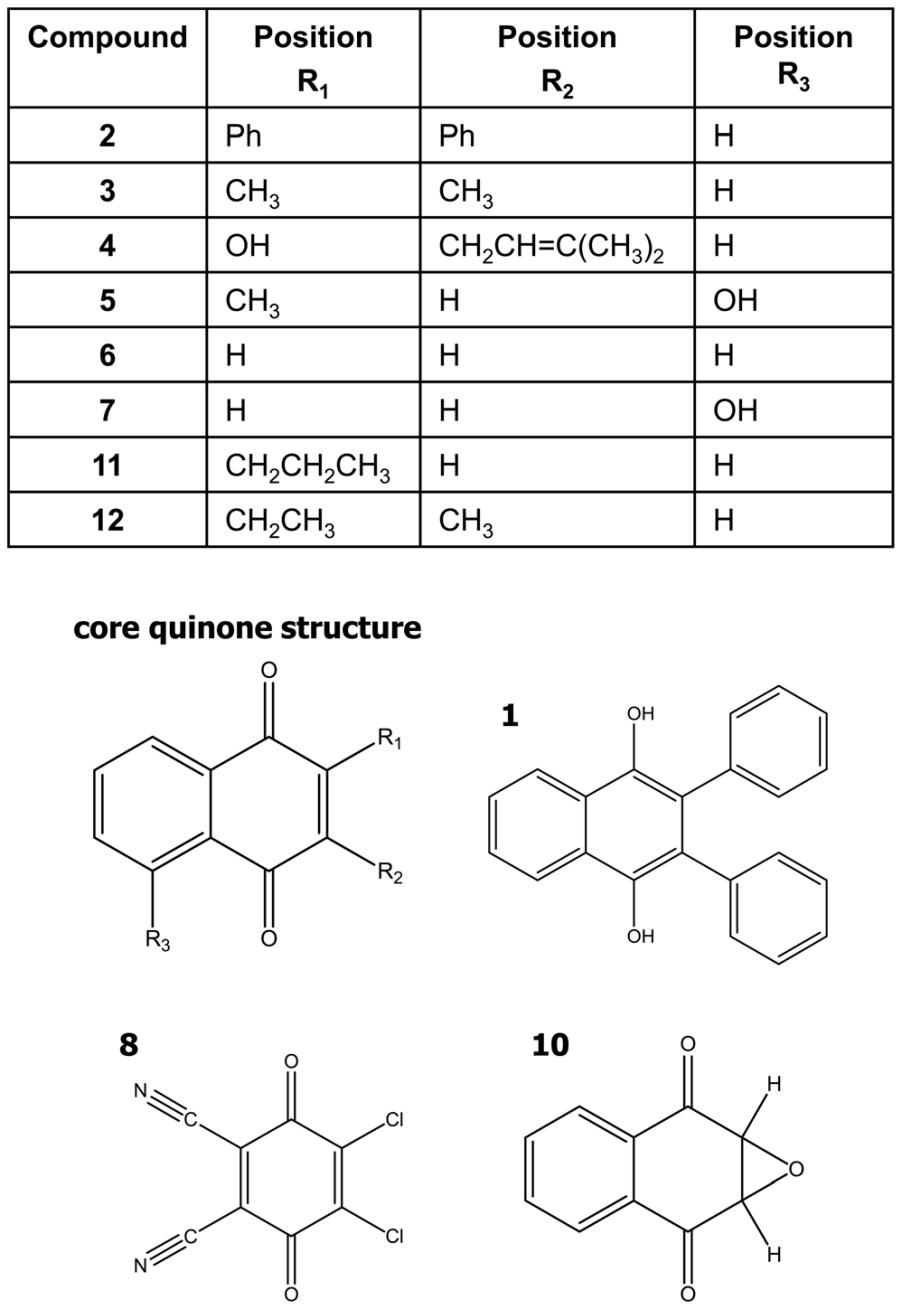
Structures of the synthetic 2, 3-disubstituted-1, 4-naphthoquinone library. All compound structures are based on the core structure (upper left), except for **1**, **8**, and **10**. Compound **9** is vitamin K1.

**Figure 2:**
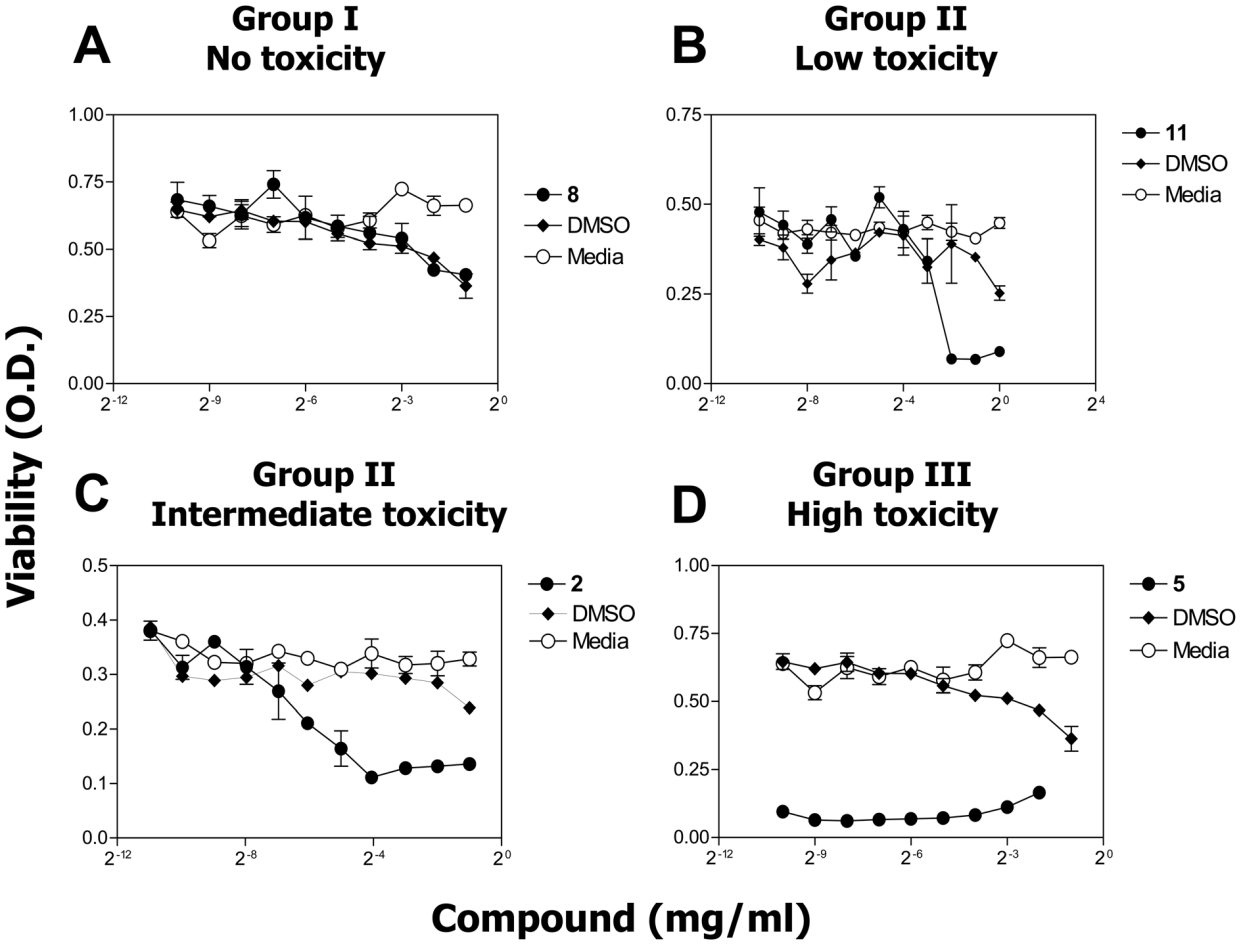
The synthetic naphthoquinone compounds vary in their ability to inhibit cellular viability. L929 cells were cultured for 48 hrs in the absence or presence of decreasing concentrations of vehicle control (DMSO) or decreasing concentrations of the indicated compound. The cells were then assessed for viability by a MTT assay. The compounds presented with no toxicity (Group I), low to intermediate toxicity (Group II), and high toxicity (Group III) (as described in the text). Compounds **2**, **5**, **8**, and **11** are representative examples of each group. The data is presented as the mean ± SEM of triplicate wells and is one of three representative experiments.

A total of twelve compounds were screened (Figure 1 and Table 1) for effects on a murine fibroblast cell line (L929). To determine if the compound library of 2, 3-disubstituted-1, 4-naphthoquinoneswere cytotoxic, the L929 cells were cultured in the presence of ten two-fold dilutions of each compound and were measured for viability. Cytotoxicity was measured as an inverse function of cell survival using a colorimetric viability assay. As indicated in Figure 2, the effects of the different compounds were placed into three groups with respect to cytotoxicity: (1) no cytotoxicity (no observed difference from DMSO vehicle control) (Fig. 2A); (2) low to intermediate cytotoxicity (the compound inhibited cell survival in a dose-responsive manner) (Fig. 2B and 2C); and (3) high cytotoxicity (>50% inhibition of cell survival at the lowest concentration examined) (Fig. 2D). The concentration of each compound that induced 50% inhibition of survival (IC_50_) is listed in Table 1. Of note are the compounds used as controls: compound **5** (plumbagin) and **7** (juglone), both which are naturally occurring. Plumbagin is found in the roots of the Plumbago plant; juglone is found in the leaves, roots and bark of plants in the Juglandaceae family, particularly the black walnut. Both compounds have been found to be quite cytotoxic in a number of model systems [15]–[17] including our L929 murine fibroblast cell line, verifying that the new synthetic scheme being used to produce the naphthoquinones accurately generates cytotoxic compounds.

**Table 1:** Summary of compounds assessed for inhibition of viability.^a^

Compound	IC_50_ b
**GROUP I – No Toxicity**	
**1**:diphenyl phenol	No inhibition
**8**:2, 3-dichloro-5, 6-dicyano-1, 4-benzoquinone	No inhibition
**9**:2-methyl-3-phytyl-1, 4-naphthoquinone (Vitamin K1)	No inhibition
**GROUP II – Low to Intermediate Toxicity**	
**2**:2, 3-diphenyl-1, 4-napthoquinone	0.030 mg/ml = 96.7 µM
**3**:2, 3-dimethyl-1, 4-napthoquinone	0.008 mg/ml = 42.9 µM
**4**:2-hydroxy-3-(3-methyl-2 butenil)-1, 4 naphthoquinone	0.015 mg/ml = 62.0 µM
**10**:1, 4-napthoquinone epoxide	0.004 mg/ml = 23.0 µM
**11**:2-propyl-1, 4-napthoquinone	0.060 mg/ml = 300 µM
**12**:2-ethyl-3-methyl-1, 4-napthoquinone	0.030 mg/ml = 150 µM
**GROUP III – High Toxicity**	
**5**:2-methyl-5-hydroxy-1, 4-napthoquinone (plumbagin)	<0.001 mg/ml = <5.3 µM
**6**:1, 4-napthoquinone	<0.001 mg/ml = <6.3 µM
**7**:5-hydroxy-1, 4-napthoquinone (jugalone)	<0.001 mg/ml = <5.7 µM

a Viability was assessed by an MTT assay at 48 hrs of incubation over a series of doubling dilutions.

b Concentration of compound that induces 50% inhibition of survival.

**Figure 3:**
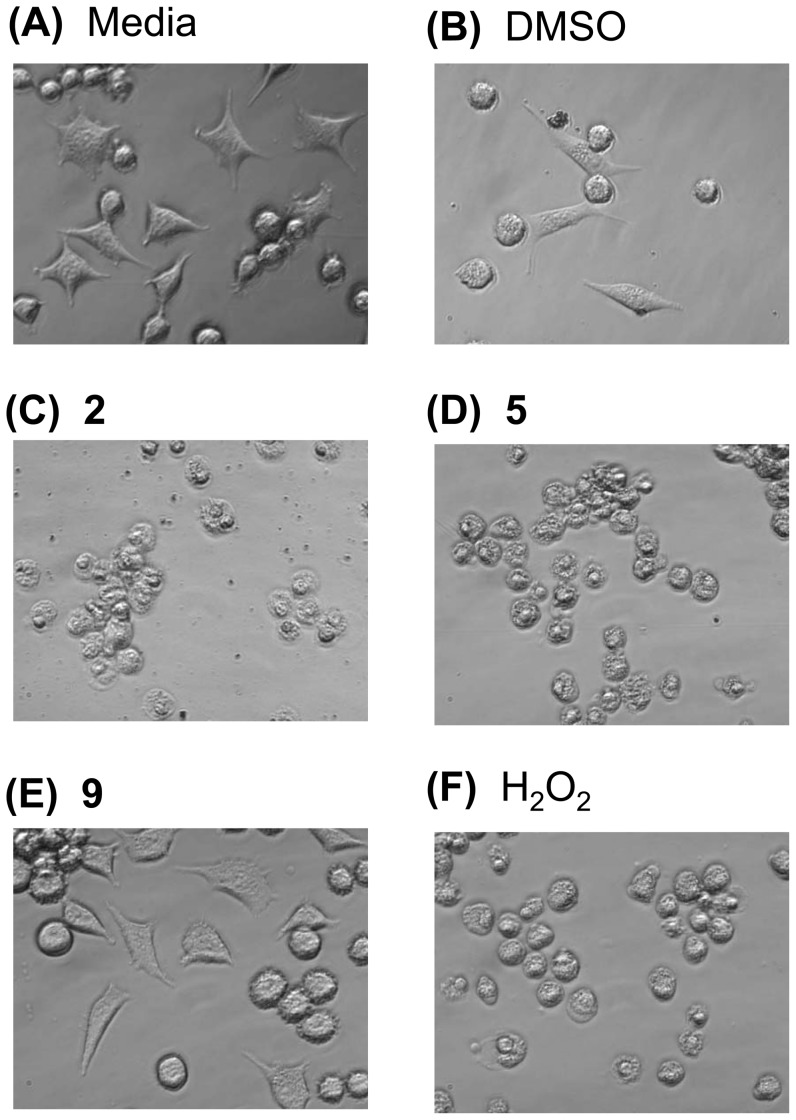
The cytotoxic compounds promote abnormal cellular morphology. The cellular morphology of L929 cells, grown in chamber well slides for 24 hrs in the presence of media alone (A), vehicle control (DMSO) (B), compound **2** (C), **5** (D), **9** (E), or H_2_O_2_ (F), was assessed by light microscopy (40 X). The cells were treated with the IC_50_ of each compound, an equivalent volume of DMSO (vehicle control), or 600 µM H_2_O_2_ (positive control).

To assess the effect of our small library of compounds on the morphology of the cells, the fibroblasts were cultured in 4-chamber well slides in the presence of each compound at the IC_50_. Following a 24 hr incubation, the slides were assessed by light microscopy. Untreated cells presented with two general morphologies: adherent-fibrous and semi-adherent rounded, which is typical of this cell line in different phases of the cell cycle (Fig. 3A). Similar morphologies were seen for cells treated with the vehicle control (Fig. 3B). In contrast, hydrogen peroxide (H_2_O_2_) treated cells were rounded, granular, and vesiculated, which is typical of dying cells (Fig. 3F). At a single concentration (IC_50_), the compounds either had no effect on the cells, as represented by compound **9** (Fig. 3E), or were detrimental to cell survival, as represented by compounds **2** and **5** (plumbagin) (Fig. 3C and 3D), further demonstrating that members of our small compound library were cytotoxic.

### Cytotoxic compounds induces phosphotidyl serine externalization and caspase 3 activation

**Figure 4:**
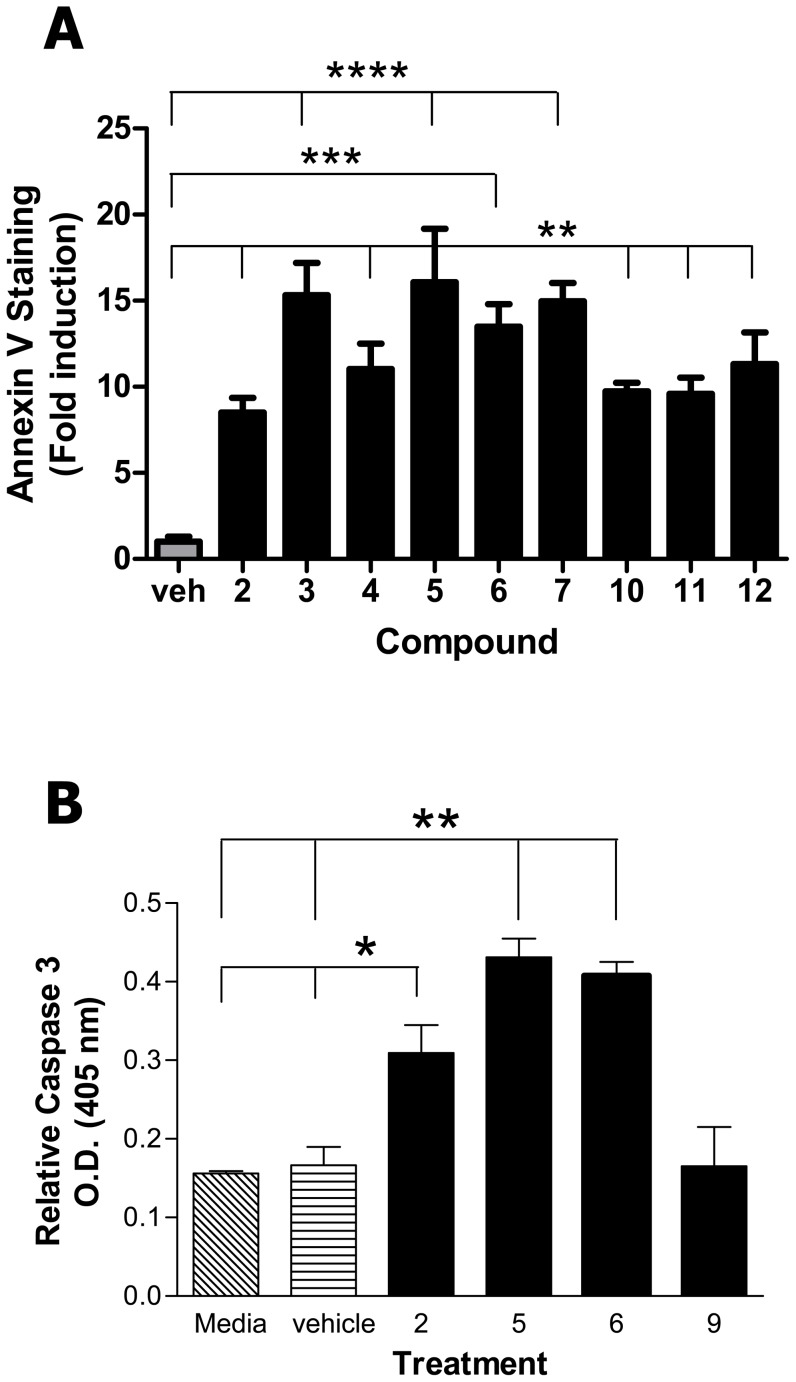
The cytotoxic compounds promote PS externalization and activate caspase 3. L929 cells were cultured for 48 hrs in the presence of media, vehicle control (DMSO), or the indicated compound (concentration  =  IC_50_) (compound **5**, plumbagin, served as an internal positive control). The cells were harvested, washed, stained with Annexin V-FITC and were assessed by flow cytometry within 30 minutes of staining (A). Percentage of apoptotic non-treated cells (background) was approximately 10–20%. The data is plotted as the fold increase in apoptosis above that induced by the vehicle control. In parallel experiments, cells grown in the presence of media, vehicle control (DMSO) or the indicated compound were assessed for the presence of active caspase 3 using a colorimetric assay kit (BioSource) (B). Although all compounds were tested, compounds **2**, **6**, and **9** are presented as representative compounds for intermediate, high, or no toxicity, respectively. Compound **5** (plumbagin) served as an internal positive control. Data is presented as the mean ± SEM of duplicate wells and is one of three representative experiments. (A) ** p≤0.001, *** p≤0.0004, **** p≤0.0001. (B) * p≤0.049, ** p≤0.0076.

We next assessed possible mechanisms the cytotoxic compounds (intermediate and high) could be utilizing to inhibit cell survival. We first assessed the loss of plasma membrane phosphatidylserine (PS) asymmetry. The appearance of PS at the cell surface, often considered a hallmark of apoptosis, has been said to prepare the dying cell to be phagocytosed [18]. We employed Annexin V staining to ascertain the extent of PS externalization upon treatment with the compounds. The L929 cells were treated with the IC_50_ for the intermediate cytotoxic compounds or with the lowest concentration tested for the highly cytotoxic compounds and were then stained with FITC-conjugated Annexin V in combination with propidium iodide (PI) and assessed by flow cytometry (Figure 4A). Very little necrosis was induced by any of the compounds (<10% above background, data not shown). In contrast, all the cytotoxic compounds (**2**, **3**, **4**, **5**, **6**, 7, **9**, **10**, and **11**) promoted externalization of PS significantly above that induced by medium alone. Compound 5, plumbagin, served as a positive control [15], [16].

Caspase 3 is thought to play a central role in the execution phase of apoptosis in that it is indispensable for several hallmark features of the process [19]. Although it has been reported that the presence of cleaved caspase 3 can be associated with non-lethal biological processes [20], the highly cytotoxic compound **5** and **7** (plumbagin and jugalone) have been shown to act through caspase 3 [21]. The ability of the cytotoxic compounds to promote activation of caspase 3 in treated L929 cells was therefore also assessed. All of the cytotoxic compounds activated caspase 3. The data is presented in Figure 4B, using compounds **2**, **6**, and **9** as representative compounds for the three cytotoxicity categories (intermediate, high, and no cytotoxicity, respectively), and compound **5** (plumbagin) as a control, Interestingly, the low/intermediate cytotoxic compounds (represented by compound **2**) activated caspase 3 equivalently to the highly toxic compounds (represented by compound **6**) and to that of the compound **5** positive control. This suggests that although caspase 3 is activated, the extent of activation was not proportional to the extent of cytotoxicity.

### Some of the cytotoxic compounds promote mitochondrial depolarization

**Figure 5:**
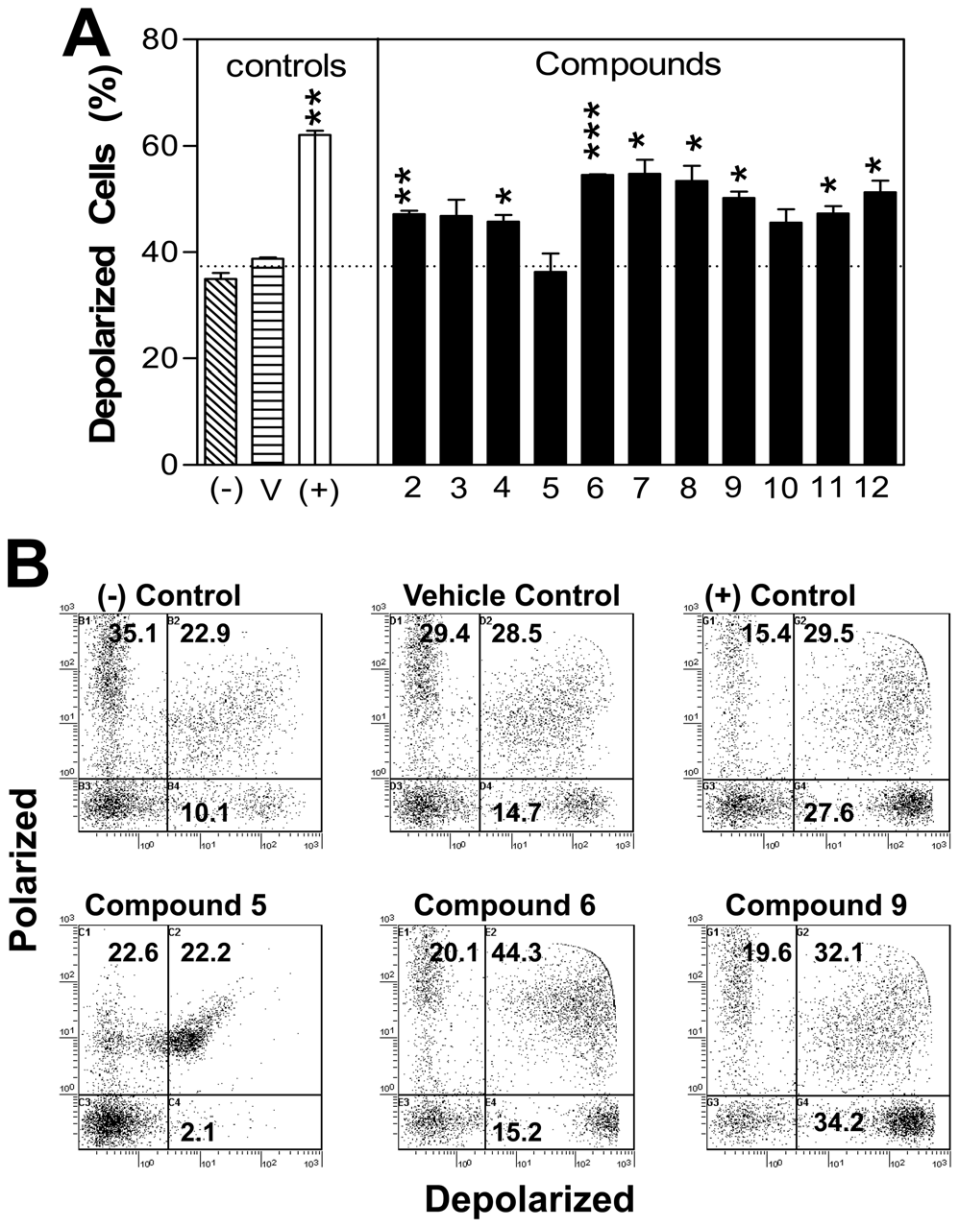
The synthetic naphthoquinone compounds vary in their ability to induce mitochondrial depolarization. L929 cells were in the presence of media (-), DMSO vehicle control (V), a positive control (+) or the indicated compound (concentration  =  IC_50_). Compound **7** (jugalone) functioned as an internal control. The cells were harvested, washed, stained with DePsipher to assess mitochondrial depolarization and were assessed by flow cytometry within 30 minutes of staining. The upper and lower right hand quadrants of the dot plots (represented in panel B) together constitute the percentage of cells that underwent mitochondrial depolarization. Data is plotted as the mean ± SEM percentage of depolarized cells from duplicate samples (A) or as representative dot plots (B). The data is taken from one of two representative experiments. * p≤0.036, ** p≤0.0066, *** p≤0.0002.

To determine if the cytotoxic compounds were promoting cell death through actions on mitochondrial membrane potential, the DePsipher Kit from Trevigen was used. DePsipher is a unique cationic dye that indicates the loss of the mitochondrial potential. The dye readily enters cells and fluoresces bright red in its multimeric form within healthy mitochondria. If the mitochondrial membrane potential were to collapse, the DePsipher reagent cannot accumulate within the mitochondria. In these cells, the reagent remains in the cytoplasm as a green fluorescent monomeric form. As shown in Figure 5A, all the cytotoxic compounds, with the exclusion of compound **5**, promoted significant changes in mitochondrial membrane potential. Compound **5** (plumbagin) is of particular interest because it is one of the most cytotoxic compounds (Fig. 2) yet its mode of action is different than any of the other cytotoxic compounds tested as is demonstrated by the flow cytometry profile (Fig. 5B).

### Cytotoxic compounds promote production of reactive oxygen species

**Figure 6:**
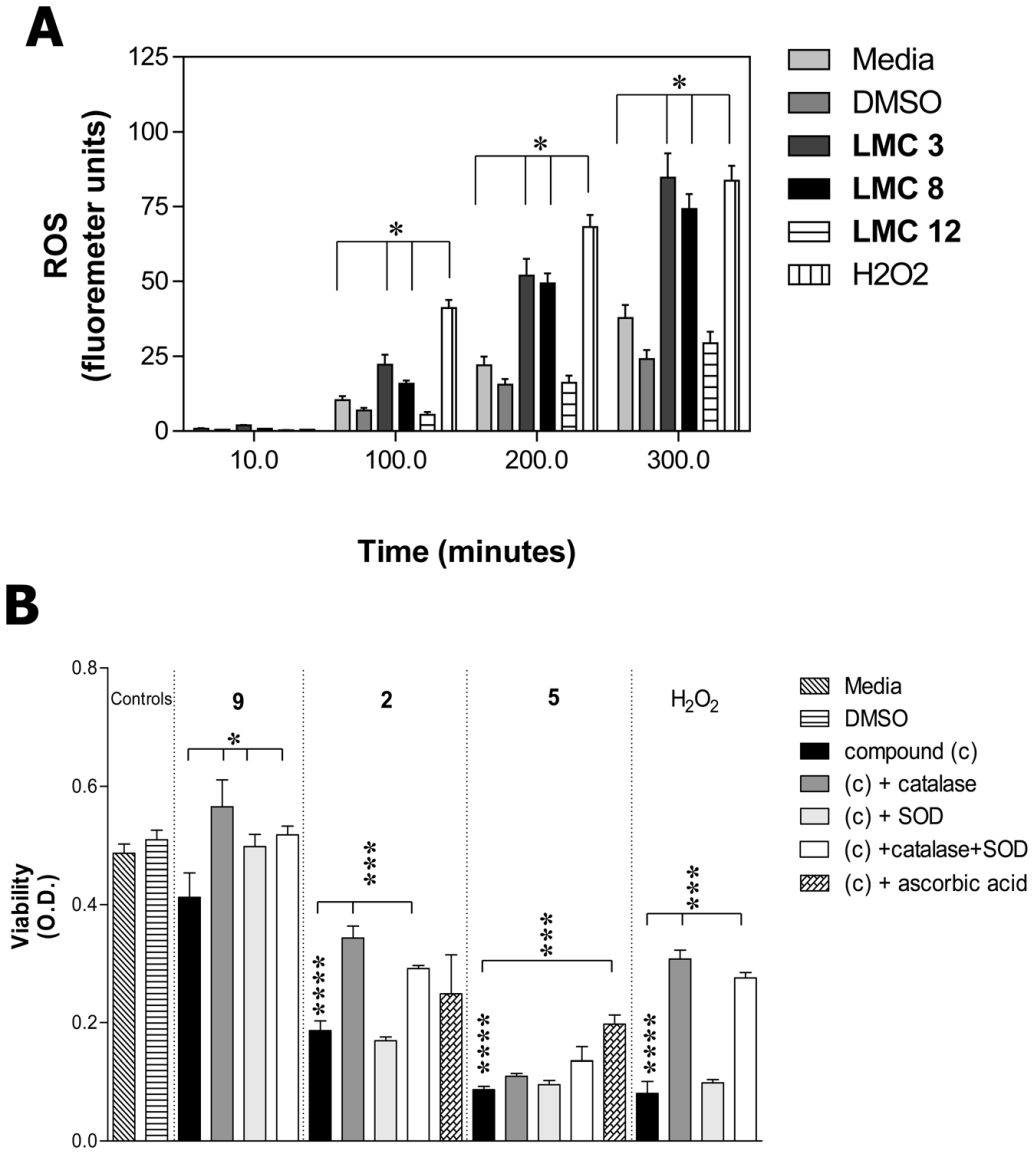
Compound-induced cell death associates with formation of ROS. L929 cells were cultured in the presence of media, vehicle control (DMSO), or the indicated compound (concentration  =  IC_50_). At 24 hrs, the cultures were assessed for the formation of intracellular reactive oxygen species (ROS) utilizing a fluorometric assay (Molecular Probes) over a 350 minute time period (A). The assay was conducted for all twelve naphthoquionones, however the only representative compounds are shown. The data is presented as the mean ± SEM of triplicate wells and is taken from one of three representative experiments. In separate experiments, the cells incubated with the compounds were also treated with catalase (1 mg/ml), superoxide dismutase (SOD, 0.5 mg/ml), or a combination of the two. A separate group of cells were pre-treated for 4 hours with 10 µM ascorbic acid, were washed and then grown in the presence of media, vehicle control (DMSO), or the indicated compound (concentration  =  IC_50_). At 48 hrs, all groups of cells were assessed for viability (B). The data is presented as the mean ± SEM of five replicate wells and is taken from one of three representative experiments. (A) * p< .001. (B). * p≤0.043, *** p≤0.0004, **** p≤0.0002, (each compound treatment, solid black, is compared to the DMSO vehicle control).

The mechanism of cytotoxicity by naphthoquinones varies due to differences in structures, diverse pharmacological effects, and different assay systems. However, the most commonly utilized mechanism appears to be the promotion of reduction-oxidation reactions [1]. The compounds are proposed to catalytically cycle and generate oxidative radicals such as hydrogen peroxide and superoxide, which then damage the cell. To assess whether our library of compounds promoted oxidative stress, L929 cells were treated with the representative compounds (**2**, **5**, and **9**) and were assessed by a fluorometric assay for the formation of reactive oxygen species (ROS) (Fig. 6A). The vehicle control (DMSO-treated) and compound **9**-treated cells induced ROS equal to that generated in non-treated cells (medium). The intermediate cytotoxic compound (**2**) and the highly cytotoxic compound (**5**) both induced levels of ROS equal to that induced by H_2_O_2_ (although not at the same rate).

To further assess the formation of oxidative radicals upon treatment with the cytotoxic naphthoquinone compounds, the compound-treated cells were concomitantly treated with the antioxidant proteins superoxide dismutase (SOD), catalase, or a combination of the two. SOD converts superoxide to hydrogen peroxide and catalase converts hydrogen peroxide to water and free oxygen, thus protecting the cells from potential ROS damage. The cells were treated with three representative compounds (**2**, **5**, and **9**) in the absence or presence of the oxidative radical inhibitors. Following a 48 hr incubation, the cells were assessed for viability.

As shown in Figure 6B, the addition of catalase partially protected compound **2**-treated cells, enhancing cell survival in the presence of the compound. The addition of SOD did not protect cells against compound **2** and the combination of catalase and SOD did not protect the cells against compound **2** beyond that provided by catalase alone. The addition of catalase, SOD, or a combination of the two also did not protect cells treated with the highly cytotoxic compound **5**. In parallel experiments, the cells to be treated with the compounds were first pre-loaded for 4 h with ascorbic acid. Ascorbic acid is a small molecule which sacrificially reacts with and eliminates oxidative radicals, and was used as a non-protein control for protection against ROS. As shown in Figure 6B, the cells treated with either compound **2** or **5** were partially protected from cell death when pre-treated with ascorbic acid. Thus, the data demonstrates that the cytotoxic naphthoquinone compounds induce oxidative radicals however, this is unlikely the only mechanism by which the compounds induce cell death.

## Discussion

Twelve 2, 3-1, 4-naphthoquinone compounds produced by a novel organic synthetic scheme were screened for cytotoxicity against a murine fibroblast cell line. Two of the compounds (plumbagin  =  compound **5** and juglone  =  compound **7**), which occur naturally in plants as chemicals for defense, were used as internal controls for synthesis of biologically relevant 1, 4-naphthoquinone derivatives. The scheme utilized was unique in that it was the first report to demonstrate the use of the Dötz benzannulation of Fischer carbene complexes with alkynes to form substituted phenols and was the first report to apply these reactions to solid-phase organic synthesis [11]. The goal of the current study was to determine if the products generated by this process possessed the biological activity commonly associated with naphthoquinones.

The small library of twelve compounds presented with three categories of cytotoxicity: no cytotoxicity, low/intermediate cytotoxicity, and high cytotoxicity. Through further analysis, the low/intermediate, and highly cytotoxic compounds were determined to promote cell death as demonstrated by membrane PS externalization and by the activation of caspase 3. Published reports have established that naphthoquinone compounds with similar structures to those of our library are commonly used in experimental models of oxidative stress induction [22]–[24]. Moreover, both plumbagin and jugalone have been shown to induce oxidative stress as well [23], [25]–[27]. We therefore sought to assess whether ROS production associated with cytotoxicity. Indeed, the formation of ROS was induced by all of the cytotoxic compounds. However, compound-treated cells were only partially protected by the anti-oxidant enzymes SOD and/or catalase or the ROS scavenger ascorbic acid suggesting the potential role of additional cell-death inducing properties of the cytotoxic compounds.

The additional cytotoxic mechanism induced by our compound library is particularly underscored by compound **5** (plumbagin), which was shown to promote the generation of ROS but does not activate mitochondrial depolarization (nor are its actions protected by SOD and/or catalase or ascorbic acid). This is in contrast to what has been seen in other mammalian cell model systems [26], [28], where changes in mitochondrial membrane potential were observed. Plumbagin may instead be inducing cell cycle arrest through effects on cell cycle progression mediators and pro-survival molecules [29], [30]. Although not tested here, it is possible that our compounds are also interacting with glutathione (GSH) or other thiol-containing proteins. Glutathione GSH, generated from the reduction of glutathione disulfide (GSSG), is a critical molecule in resisting oxidative stress acting as a scavenger for hydroxyl radicals, singlet oxygen, and various electrophiles [31], [32]. In this capacity, it is possible that the electrophilic disubstituted 1, 4-naphthoquinone compounds interact with GSH inhibiting its ability to scavenge ROS. This may explain the partial protection from ROS formation imparted by SOD, catalase, and ascorbic acid upon treatment with the compounds. Indeed, quinones and naphthoquinones, including jugalone, have been shown to readily cause depletion of intracellular GSH [23], [24], [33], [34].

In summary, our small library of compounds has yielded a group of synthetically generated naphthoquinones that mediate cytotoxicity. Induction of cell death by the cytotoxic compounds is associated with PS externalization, activation of caspase 3, and induction of ROS formation. Our data is in agreement with reports demonstrating that compounds with structures similar to our compounds are cytotoxic and induce cellular oxidative stress [22]–[29]. Of significance is that in our study, 75% of the synthetic compounds possess pro-cell death activity as determined by a number of assays in a well-defined eukaryotic cell system, offering a number of reagents for future study and highlighting the effectiveness of the unique organic synthetic scheme to generate biologically active naphthoquinone compounds.
