# Correction: A Small Library of Synthetic Di-Substituted 1, 4-Naphthoquinones Induces ROS-Mediated Cell Death in Murine Fibroblasts

**DOI:** 10.1371/journal.pone.0116133

**Published:** 2014-12-16

**Authors:** The PLOS ONE Staff

Drs. Abril Estrada, Qingyi Li, and Luis Martinez should be included in the author byline. They should be listed as the fourth, fifth, and sixth, authors respectively.

Dr. Estrada’s affiliation is 3: The Clorox Services Company, Pleasanton, California, United States of America. The specific contributions of this author are as follows: Contributed reagents/materials/analysis tools, synthesized the compounds that were tested.

Dr. Li’s affiliation is 4: Discovery Chemistry, Cubist Pharmaceuticals, Lexington, Massachusetts, United States of America. The specific contributions of this author are as follows: Contributed reagents/materials/analysis tools, synthesized the compounds that were tested.

Dr. Martinez’s affiliation is 5: Center for Innovation and Entrepreneurship, Trinity University, San Antonio, Texas, United States of America. The specific contributions of this author are as follows: Conceived and designed the experiments, contributed reagents/materials/analysis tools, established the collaboration.

The correct citation is: Ramirez O, Motta-Mena LB, Cordova A, Estrada A, Li Q (2014) A Small Library of Synthetic Di-Substituted 1, 4-Naphthoquinones Induces ROS-Mediated Cell Death in Murine Fibroblasts. PLoS ONE 9(9): e106828. doi:10.1371/journal.pone.0106828
